# A Microfluidic, Flow-Through, Liquid Reagent Fluorescence Sensor Applied to Oxygen Concentration Measurement

**DOI:** 10.3390/s23104984

**Published:** 2023-05-22

**Authors:** Dominik Gril, Denis Donlagic

**Affiliations:** Laboratory for Electro Optics and Sensor Systems, Faculty of Electrical Engineering and Computer Science, University of Maribor, Koroska Cesta 46, 2000 Maribor, Slovenia

**Keywords:** microfluidic, microfluidic sensing systems, fluorescent sensors, oxygen sensing, fluorescent decay time measurements, on-line liquid analysis, optical fibers, capillaries, liquid reagent

## Abstract

A concept of a microfluidic fluorescent chemical sensing system is presented and demonstrated as a sensor for measurement of dissolved oxygen in water. The system utilizes on-line mixing of a fluorescent reagent with the analyzed sample, while it measures the fluorescence decay time of the mixture. The system is built entirely out of silica capillaries and optical fibers, and allows for very low consumption of the reagent (of the order of mL/month) and the analyzed sample (of the order of L/month). The proposed system can, thus, be applied to continuous on-line measurements, while utilizing a broad variety of different and proven fluorescent reagents or dyes. The proposed system allows for the use of relatively high-excitation light powers, as the flow-through concept of the system reduces the probability of the appearance of bleaching, heating, or other unwanted effects on the fluorescent dye/reagent caused significantly by the excitation light. The high amplitudes of fluorescent optical signals captured by an optical fiber allow for low-noise and high-bandwidth optical signal detection, and, consequently, the possibility for utilization of reagents with nanosecond fluorescent lifetimes.

## 1. Introduction

Fluorescence sensing is an established approach in chemical and biochemical sensing. It frequently utilizes fluorescent dyes or other suitable reagents, which react to the presence of an analyte by modulating their fluorescent decay times [1,2,3,4,5,6,7,8,9,10]. When this very versatile approach is applied to sensor design and on-line measurements, fluorescent dyes/reagents are often immobilized, or incorporated into different thin films of other layers. Molecules of the analyte penetrate the surface of these thin films or layers, and interact with the immobilized fluorescent dye/reagent to modulate their decay times. The incorporation of fluorescent dyes/reagents into porous materials or polymer matrices is thus a common approach in building fluorescence sensors [1,3,4,11,12,13,14,15,16,17,18,19,20,21,22,23]. The immobilization of fluorescent dyes or reagents in thin films or active layers, however, brings several limitations, which are often reflected in long-term measurement system zero-point drifts, hysteresis, limited signal amplitude/quality, and film material compatibility issues with operating environments. Other limitations associated with the incorporation of reagents into thin films are also often associated with dye/reagent bleaching, permanent sensing film contamination, sensing film swelling, low fluorescence signal yields as the volume/thickness of the active region is limited to the penetration depth of the analyte into the active layer, slow response time, and the requirement for reversibility of the chemical reaction between the dye/reagent and the analyte [11,24,25,26,27,28].

An alternative approach to the dye/reagent immobilization utilizes direct mixing of an analyte with the dye/reagent, followed by optical analysis of the mixture [29,30,31,32]. While this approach is often used in laboratory environments, it is generally considered less suitable for on-line control applications, as it consumes significant quantities of the dye/reagent, generates a substantial amount of waste—which can be problematic for disposal—and is difficult for realization in fully autonomous setups.

This paper presents a silica capillary-based microfluidic system that utilizes direct and continuous mixing of dye/reagent with the analyzed liquid, while providing very low consumption of reagent and the analyzed sample. This reduces the amount of generated waste products to a level that will be acceptable for many continuous/on-line process monitoring applications. The proposed system also maximizes the volume of the optically excited sample–reagent mixture, and thus provides favorable geometry for high intensity of excitation light and efficient fluorescent light collection by the application of optical fibers. The latter provide an opportunity to generate and collect a substantial amount of fluorescent light, which further allows the use of a relatively small surface area, and high-speed optical detectors. This yields the possibility for a design of a high electrical bandwidth optical measurements system that can allow for reliable measurements of short fluorescence decay times. The latter opens a variety of different sensing opportunities, as short decay time dyes/reagents cover a broad range of different analytes [33,34,35,36,37,38,39]. The proposed microfluidic setup was demonstrated as an on-line sensing system for the measurement of dissolved oxygen in water, using two different fluorescent dyes with decay times between 400 ns and 1 µs.

## 2. Microfluidic, Flow-Through Fluorescence Design

**Figure 1:**
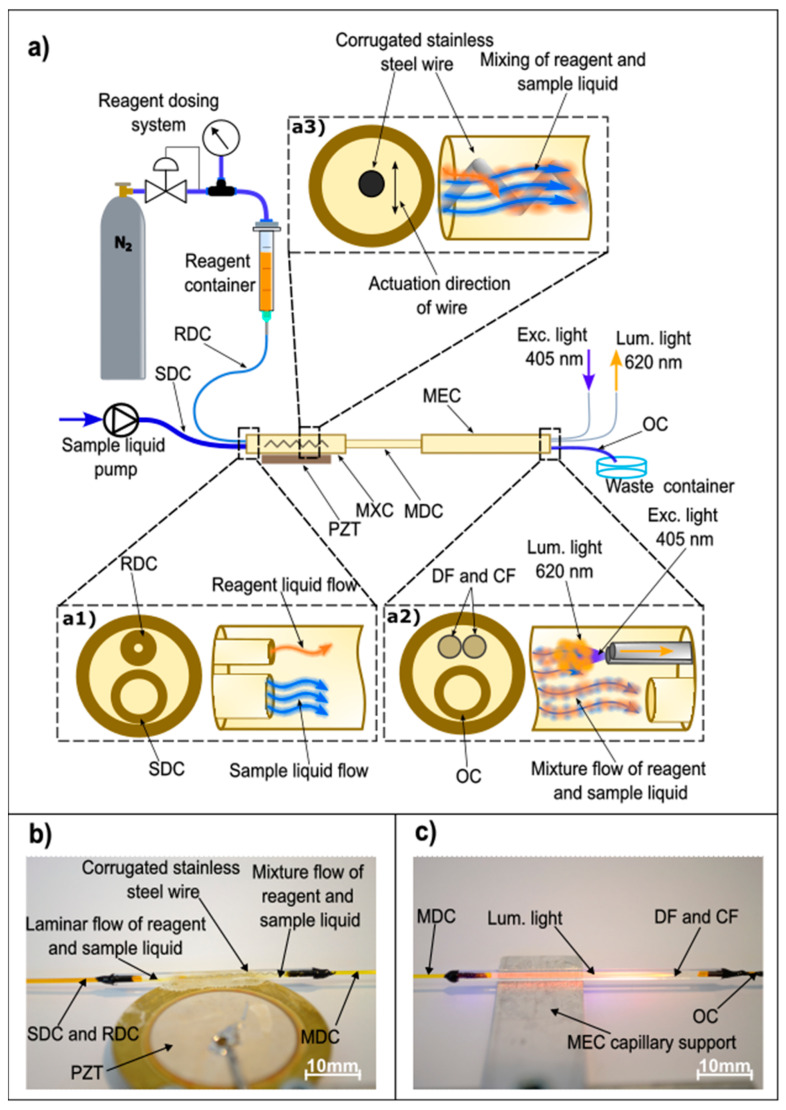
(**a**) Microfluidic, flow-through, liquid reagent fluorescence sensing system (RDC-reagent delivery capillary, SDC-sample delivery capillary, OC-output capillary, MXC-mixing capillary, MDC-mixture delivery capillary, MEC-measurement capillary, DF-delivery fiber, CF-collecting fiber, PZT-piezo driven actuator, Exc. Light-excitation light, Lum. Light-luminescent light). (**a1**) Detailed schematic of sample and reagent inlet. (**a2**) Detailed schematic of liquid outlet, light excitation and light collection. (**a3**) Detailed schematic of sample and reagent mixing. (**b**) Mixing capillary mounted on a PZT actuator (RDC-reagent delivery capillary, SDC-sample delivery capillary, MDC-mixture delivery capillary, PZT-piezo driven actuator). (**c**) Measurement capillary during operation (MDC-mixture delivery capillary, OC-output capillary, DF-delivery fiber, CF-collecting fiber).

The proposed system’s scheme is shown in Figure 1, and was built out of silica capillaries and a pair of optical fibers.

The liquid to be analyzed (oxygenated water in our particular demonstration case) was pumped into a sample delivery capillary (SDC), which had inner and outer diameters of 320 µm and 390 µm, respectively. The applied micro-pump provided a constant volumetric flow of the analyzed liquid. Another reagent delivery capillary (RDC), with a smaller inner diameter corresponding to 25 µm, and length of 23.5 cm, was connected to a container with the dye/reagent. The container was pressurized by nitrogen gas. The pressure was adjustable by a gas pressure regulator within a range from 0 to 5 bar. Both capillaries were inserted into a larger, mixing capillary (MXC), with inner and outer diameters of 1000 µm and 2000 µm, respectively, as shown by insert A1 in Figure 1. The mixing capillary had a length of 35 mm and a mixture delivery capillary (MDC) connected to its output side. The mixture delivery capillary had inner and outer diameters of 530 µm and 650 µm, respectively, and a length of 110 mm. Furthermore, a 150 µm diameter corrugated stainless steel micro-wire with a length of 20 mm was inserted into the mixing capillary, while the mixing capillary was fixed onto a PZT driven actuator (Bimorph™). The mixture delivery capillary was, further, fed into a measurement capillary (MEC) with an inner diameter of 1000 µm and an outer diameter of 2000 µm. At the output of the 47 mm long measurement capillary, two optical fibers and an output capillary (OC) were inserted into the measurement capillary, as shown by insert A2 in Figure 1. Both fibers and capillary were placed in a parallel geometric configuration, as shown in Figure 1. The first, delivery fiber (DF), was used to deliver the excitation light into the measurement capillary, while the second, collection fiber (CF), was used to collect and guide the fluorescent light to an opto-electronic interrogation system. Both fibers had 125 µm claddings, 105 µm cores, and a numerical aperture of NA = 0.22. The output capillary had inner and outer diameters of 320 µm and 390 µm, respectively, and was 200 mm long. It was used to dispose of the fluid into a waste fluid collection container. The proposed capillary system was assembled experimentally under an optical microscope using linear motion stages for positioning. All the joints between the capillaries and fibers were sealed by higher viscosity epoxy (3M Scotch-Weld, DP490) and cured at 80 °C to form a stable structure. The all-capillary design provided a straightforward sensor system assembly, while it assured easy connection of the system to the sources of liquids and the optical source and detector. The proposed system thus eliminates the need for complex packaging that is typical for lab-on-a-chip and similar solutions. Examples of the mixing and measurement capillary sections of the produced sensor are also shown in Figure 1b,c. Assembly of the capillaries is straightforward, and can take advantage of tools and processes that are known from optical fiber production, preparation, and handling. Fiber optic assemblies of comparable complexity are produced nowadays in high volumes. Gas cylinders can be replaced by commercial micropumps.

The mixing, mixture delivery, measurement, and output capillaries had considerably larger cross-sections and shorter lengths than the reagent delivery capillary, which guaranteed that the pressure at the output of the reagent delivery capillary was always close to atmospheric pressure. The relatively small diameter of the reagent delivery capillary and the relatively high drive pressure used in the reagent container ensured that an almost entire pressure drop developed over the reagent delivery capillary. Therefore, the applied pressure across the capillary ∆*P*, the reagent viscosity *η*, capillary radius *r* and length *l* defined the volumetric flow *Q* of the reagent into the mixing capillary, which can be approximated as:

(1) $$Q=\frac{\Delta P\times r^{4}}{\frac{8}{\pi }\times \eta \times l}$$

Pressure regulation in the reagent container thus allowed for simple control over the reagent flow rate. The sample fluid was pumped by a syringe pump during the experimental investigation. However, for in-the-field applications, micro-pumps would need to be used for continuous extraction of a sample from the monitored system. The syringe pump also provided possibilities for an easy and rapid exchange of reference test samples with different analyte concentrations.

**Figure 2:**
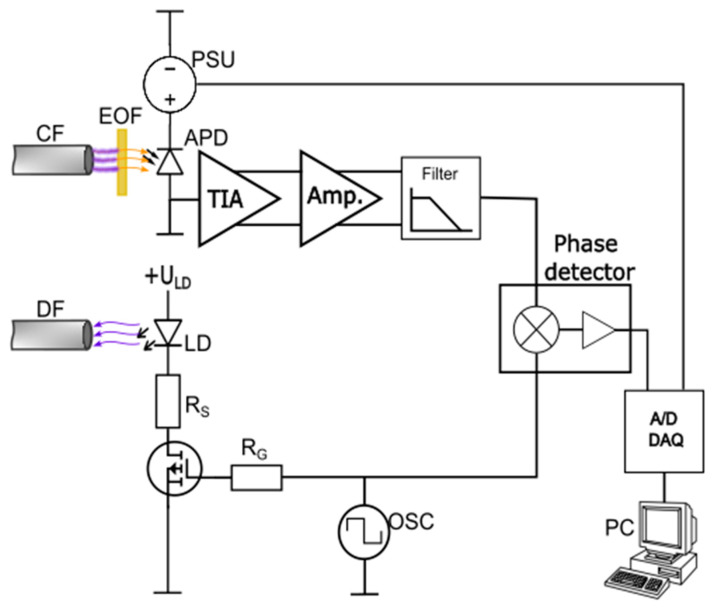
Signal interrogation system: TIA-Trans impedance amplifier (AD8015), Amp.-amplifier (LTC1992-5), Filter-4th order low pass filter (LT6600-2.5), Phase detector (AD8302), APD-avalanche photo diode (C30902EH), LD-laser diode, OSC-electrical oscillator, PSU-programmable power supply, CF-collecting fiber, DF-delivery fiber, EOF-edge pass optical filter, DAQ-Data acquisition system, PC-personal computer with LabView, Rs-current limiting resistor, RG-gate resistor).

The signal interrogation system, shown in Figure 2, consisted of an electrical oscillator with a programmable frequency, a laser driver, a laser diode, an avalanche photodetector (APD), a programmable high voltage source, a low-noise transimpedance amplifier (TIA), a bandpass filter, a phase detector and a personal computer (PC) with a data acquisition card and LabView software package for data analysis. In the current experimental setup we used a 405 nm fiber-coupled laser diode for excitation.

To demonstrate the operation of the proposed microfluidic system, we applied the system to measurement of dissolved oxygen in water. For this purpose, we used known oxygen-quenching dyes, [Ru(bpy)_3_]^2+^2Cl^−^ and [Ru(phen)_3_]Cl_2_, detailed information in Table A1. These dyes were selected as they are cost-efficient, easily available/accessible, and, since they are compatible with broadly available, cost-efficient, 405 nm GaN laser diodes. Furthermore, these dyes are highly water-soluble, which allows for preparation of highly concentrated liquid reagents, that minimizes reagent consumption further. It should also be stressed that the proposed microfluidic system can be applied to other fluorescent reagents for detection of oxygen or other parameters of interest, and is not limited to the aforementioned dyes. The main motivation for this work was to demonstrate a complete and versatile microfluidic system that can be used with a variety of different reagents and analytes, not just dissolved oxygen detection.

The oscillator frequency was adapted to a particular reagent/dye fluorescent time, to maximize and linearize the phase change in response to variations in the fluorescent time of the analyzed mixture. For example, as discussed further below, a 700 kHz excitation frequency was applied in the case of the 400 ns fluorescent decay time dye ([Ru(bpy)_3_]^2+^2Cl^−^), and to 350 kHz in the case of a 1000 ns fluorescent decay time dye ([Ru(phen)_3_]Cl_2_). Furthermore, an edge optical filter with an edge wavelength of 450 nm was placed between the APD and the collection fiber, to eliminate further excitation light from the fluorescence signal. This required the application of a larger-area APD detector. In our case we used an APD with a diameter of 0.5 mm, produced by Excelitas Technologies. The laser driver was realized with a MOSFET transistor and a current limiting resistor, which generated a nearly square-shaped current through the laser diode. The APD was supplied by about 220 V of reverse-bias voltage, and was connected to a low-noise telecom TIA (AD8015). The output of the TIA was amplified further by an amplifier and filtered with a 4th order Chebyshev filter, with a cutoff frequency of 2.5 MHz. The amplified and filtered signal from the APD was then fed to the integrated phase detector (AD8302), while using the output of the oscillator as a reference. AD8302 [40] compares two electrical signals and converts the phase difference between these signals into a voltage. We used the manufacturer-recommended configuration of AD8302, which yielded an AD8302 output phase sensitivity of S_P_ = 10 mV/°. A time-constant change was then obtained by multiplying the phase detector output voltage by S_P_/(360°*f), i.e., Δ$\tau =\frac{U*S_{P}}{360{}^{\circ}\cdot f}$ (where *f* is the excitation frequency and *U* the voltage at the AD8302 output). The voltage output of the AD8302 (which was proportional to the phase difference between the excitation and the fluorescent signal) was then connected to the personal computer through a data acquisition card. The LabView instrumentation software package was used for further processing and data display. The voltage measured at the output of the AD8302 was multiplied by the constant that relates the voltage and phase difference (as defined in the AD8302 specifications). By multiplying the phase difference further by 1/2π, we obtained the fluorescence lifetime, which correlated directly with the analyte concentration.

**Figure 3:**
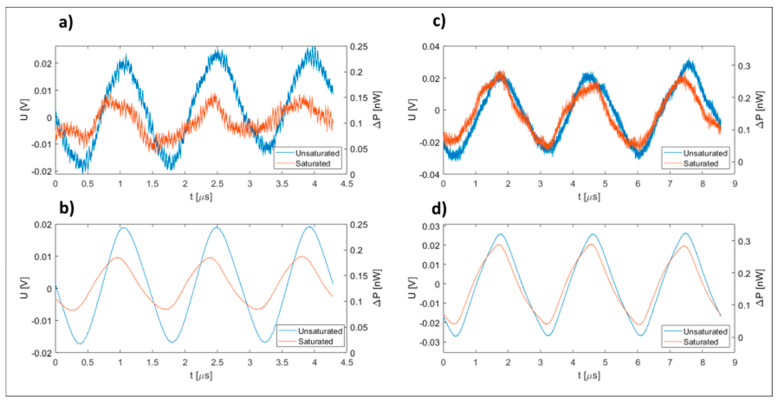
Electrical signals recorded by the oscilloscope at the input of the phase detector: (**a**) Raw signal, (**b**) Averaged signal (128 samples) for the [Ru(bpy)_3_]^2+^2Cl^−^ dye; (**c**) Raw signal, (**d**) Averaged signal (128 samples) for the [Ru(phen)_3_]Cl_2_ dye. The differently colored curves represent the two extreme values obtained when applying lean and full oxygen-saturated water to the proposed sensing system.

The proposed system was set up for the measurement of dissolved oxygen concentration in water. Two different oxygen-quenching dyes were selected: [Ru(bpy)_3_]^2+^2Cl^−^ (Ruthenium-tris(2,2′-bipyridyl) dichloride) and [Ru(phen)_3_]Cl_2_ (Dichlorotris(1,10-phenanthroline)ruthenium(II) chloride). While both selected dyes are similar in molecular structure, the first selected dye provided a strong change in the fluorescence time at a higher oxygen concentration [41,42,43,44], while the second dye provided a more uniform response across the entire range of oxygen concentrations, and was used to demonstrate system operation at low oxygen concentrations. The test reagents were prepared by dissolving 1 wt% concentrations of [Ru(bpy)_3_]^2+^2Cl^−^ and 0.1 wt% of [Ru(phen)_3_]Cl_2_ in demineralized and degassed water, respectively. The prepared reagents were filtered further, and placed in light non-transparent reagent containers. The reagent flow was set/controlled between 5.8 nL/min and 35 nL/min by adjusting the drive nitrogen gas pressure between about 0.2 and 1.4 bar, as discussed in the Experimental section below. The sample flow was set to a considerably higher flow rate of 58 µL/min, and was kept constant during all the experiments. A Bimorph PZT actuator with mounted capillary was set in a resonance mechanical motion in a direction perpendicular to the mixing capillary’s longitudinal axis by application of a 170 Hz sinusoidal drive voltage, which caused the mixing capillary to oscillate with a peak-to-peak amplitude of about 0.4 mm. The PZT-induced vibrations, and the corrugated microwire packaged within the mixing capillary, caused efficient mixing of the reagent and the sample, which does not normally occur within capillaries of the given sizes. The laser driver and the excitation laser diode were set to generate about 15 mW of average power at the output of the delivery fiber. The 405 nm light delivered into the measurement capillary excited the sample–reagent mixture, while the generated fluorescent light was acquired by the collection fiber. Since the excitation light was scattered within the measurement capillary, a partial recapture of excitation light by the light collection fiber was unavoidable, and the optical filter (as already described above) was, thus, necessary, to remove the excitation (405 nm) component from the light captured by the collection fiber. The filtered (fluorescent) average optical power, acquired and measured at the output of the collection fiber, corresponded to about 20 nW with the [Ru(bpy)_3_]^2+^2Cl^−^ dye, and approximately 15.3 nW for the [Ru(phen)_3_]Cl_2_ dye (using the oxygen-depleted water sample), which is a relatively high fluorescent power suitable for subsequent high-speed opto-electronic signal processing. The insertion of the optical filter between the fiber and detector required an increase in distance between the detector and fiber, which required a larger-area detector. A more optimal configuration could likely be obtained by the creation of an in-line fiber filter, which was not available commercially during the implementation of this work. While an increase in APD detector size yields a degradation of the system’s signal-to-noise ratio (SNR), the final SNR after filtering still corresponded to 14 dB in the case of the [Ru(bpy)_3_]^2+^2Cl^−^ dye and 17 dB in the case of the [Ru(phen)_3_]Cl_2_ dye. Figure 3 shows the raw and averaged (128 averages) signals as recorded by the oscilloscope at the input to the phase detector. The obtained SNR was more than adequate to obtain reliable and low noise operation of the phase detector.

## 3. Experimental Results

The above-described system was evaluated by a series of experiments. Glass syringes filled with demineralized water and having different concentrations of oxygen (ranging from 0 to 34 mg/L) were prepared at room temperature, and were exchanged manually within the syringe pump to generate sample flows through the sensor with different oxygen concentrations. The concentration of oxygen in the water solution in each prepared syringe was measured shortly prior to the test measurement by a reference dissolved oxygen laboratory instrument, produced by Anton Paar, model OxyQc Wide Range.

**Figure 4:**
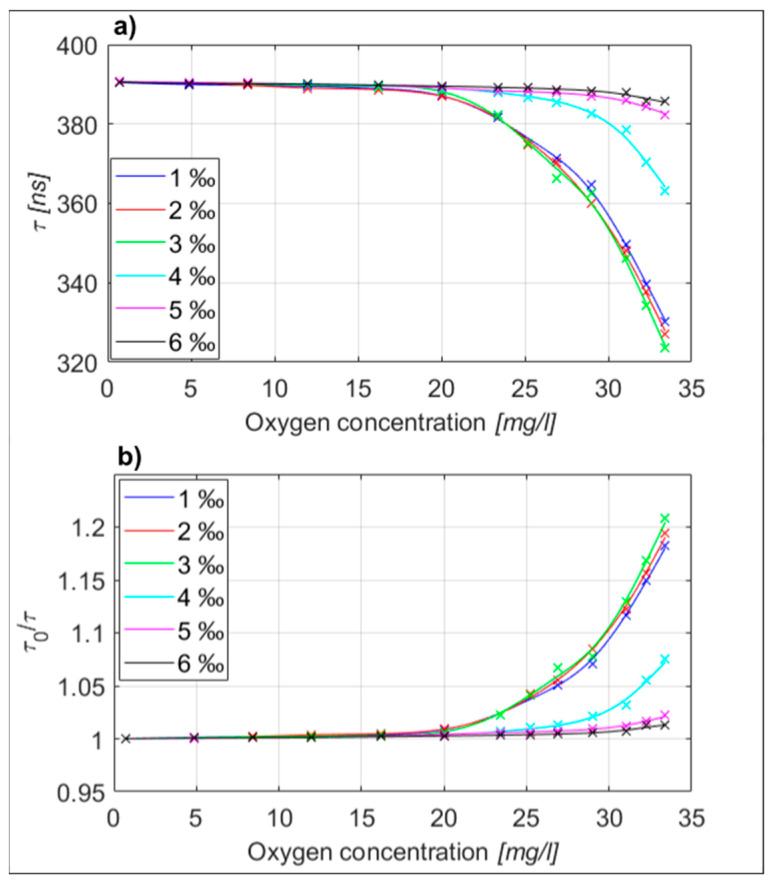
(**a**) Measured lifetime for different reagent concentrations. (**b**) Normalized lifetime for different reagent concentrations.

Firstly, multiple static characteristics (the phase shift between the excitation and fluorescent signal versus dissolved oxygen concentration) were measured at different reagent flow rates, as shown in Figure 4. In this experiment static characteristics were measured by applying water with different dissolved oxygen concentrations to the test system, while repeating the test multiple times by using different reagent flow rates. The results in Figure 4 indicate that an optimum reagent flow rate exists for the given reagent concentration and sample flow rate, which is likely a consequence of the optimum stoichiometric conditions that need to be met for the best static response. In our particular case the steepest static characteristics were obtained when the reagent flow (using a reagent with 1 wt% [Ru(bpy)_3_]^2+^2Cl^−^ dye in water) and analyte flow rate were in a 0.3:1000 ratio. This experiment was conducted only for the reagent using 1 wt% [Ru(bpy)_3_]^2+^2Cl^−^ dye.

**Figure 5:**
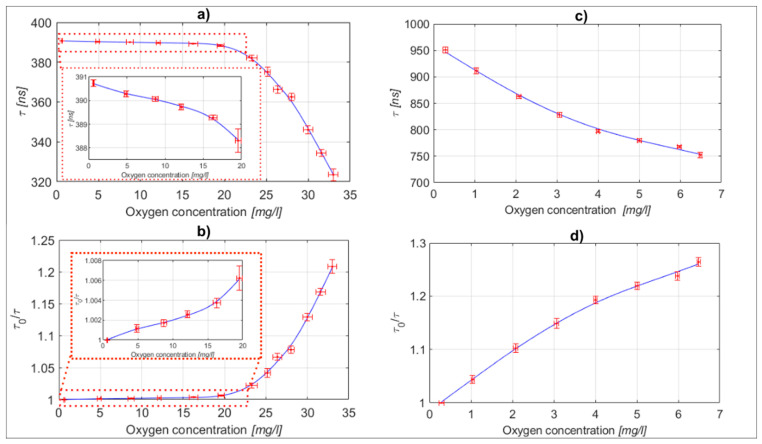
Static characteristic with confidence intervals: (**a**) Measured lifetime vs. oxygen concentration for the sensing system using the [Ru(bpy)_3_]^2+^2Cl^−^ dye. (**b**) Normalized lifetime vs oxygen concentration for the sensing system using the [Ru(bpy)_3_]^2+^2Cl^−^ dye (**c**) Measured lifetime vs oxygen concentration for the sensing system using the [Ru(phen)_3_]Cl_2_ dye. (**d**) Normalized lifetime vs oxygen concentration for the sensing system using the [Ru(phen)_3_]Cl_2_ dye.

After the initial flow rate optimization was performed, we measured multiple static characteristics to assess the repeatability and measurement uncertainty of the proposed measurement system. All the experiments were carried out at a constant reagent-to-sample flow rate, which corresponded to a 0.3:1000 ratio. In the case of [Ru(bpy)_3_]^2+^2Cl^−^ reagent use, we prepared 13 different water samples, which covered oxygen concentrations from 0 to over 34 mg/L (which is also about the maximum stable oxygen concentration in water at room temperature). This allowed evaluation of the system’s response over the entire static characteristic, which was measured 10 times to establish the confidence intervals. The results are shown in Figure 5, and demonstrated relatively non-linear and “steep” static characteristics in the range of higher concentrations (>20 mg/L), which is typical of [Ru(bpy)_3_]^2+^2Cl^−^ dye, as, for example, demonstrated in [10,45]. To demonstrate the operation of the proposed system at lower and more commonly encountered oxygen concentrations in water, we used the [Ru(phen)_3_]Cl_2_ reagent. For this test we prepared eight different water samples distributed across a concentration range between 0 and 6.5 mg/L. The static characteristics’ measurement, shown in Figure 5, was also repeated 10 times to obtain the confidence intervals. The measured static characteristic for [Ru(phen)_3_]Cl_2_ dye showed a considerably higher tendency to linear concentration response in comparison to the [Ru(bpy)_3_]^2+^2Cl^−^ dye, but provided lower sensitivity at higher oxygen concentrations. [5,8,9,10]. The oxygen concentration of each sample was controlled by the reference instrument (as described above) prior to each individual test. From the conducted tests the measurement uncertainty of the system can be estimated at 0.85 mg/L for the [Ru(bpy)_3_]^2+^2Cl^−^ reagent in a water sample concentration range above 20 mg/L, while the [Ru(phen)_3_]Cl_2_ regent yielded an uncertainty of about 0.12 mg/L in the sample range of 0–6 mg/L (which is also close to the declared uncertainty of the used reference instrument, and might, thus, be even higher, as we were limited by the performance of the used reference instrument).

**Figure 6:**
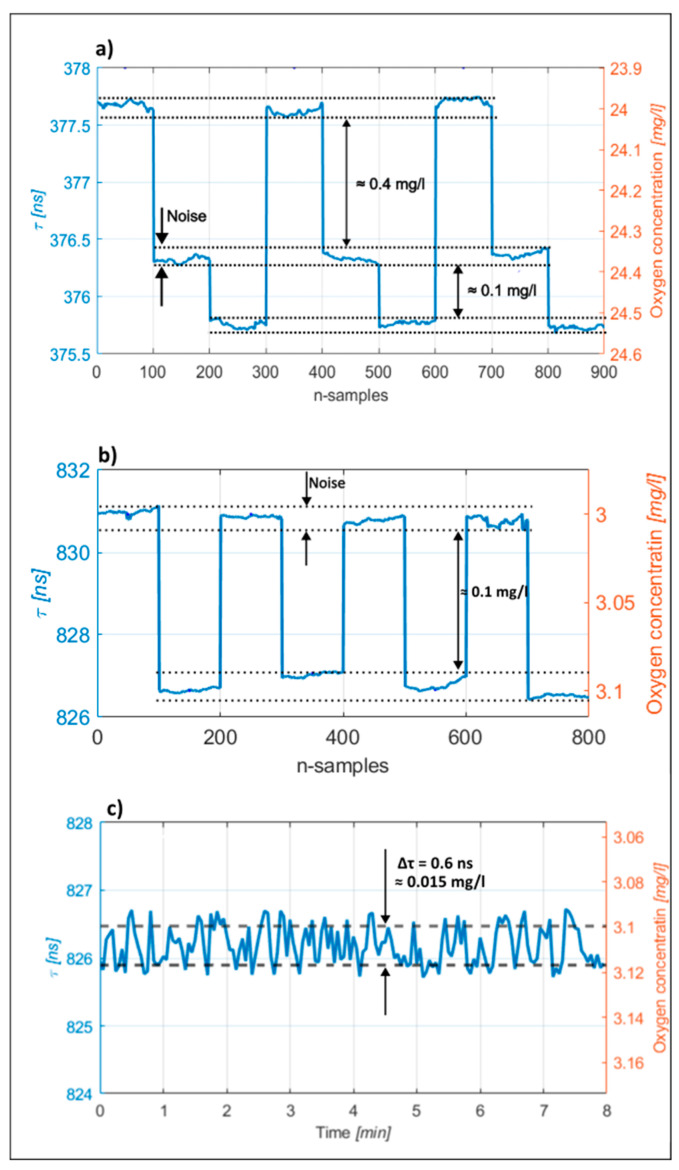
Resolution tests: (**a**) System response to periodic variation of the oxygen concentration for the [Ru(bpy)_3_]^2+^2Cl^−^ dye reagent. The base concentration of about 24 mg/L was firstly increased by 0.4 mg/L, and then by an additional 0.1 mg/L (i.e., to 24.6 mg/L); (**b**) Periodic variation of the oxygen concentration for the [Ru(phen)_3_]Cl_2_ dye reagent, base concentration of about 3 mg/L was increased by 0.1 mg/L to about 3.1 mg/L and then decreased back to 3 mg/L; the process was repeated periodically. (**c**) Noise at the output of the measurement over a longer period of time (around 8 min); the noise level was about of 0.6 ns, which corresponds to an oxygen concentration change of 0.015 mg/L.

A series of tests were also conducted to evaluate the achievable measurement resolution limits for the proposed system. A series of syringes, which possessed samples with small differences in oxygen concentrations, were prepared for this experiment. These syringes were connected/interchanged to the sample delivery capillary interchangeably/periodically, while observing the measured output voltage/phase change. For the [Ru(bpy)_3_]^2+^2Cl^−^ regent, the resolution test was performed at a base oxygen concentration of 24.4 mg/L. Similarly, for the system with [Ru(phen)_3_]Cl_2_, the base concentration for conducting the resolution test corresponded to 3 mg/L. The signal provided by the phase detector was filtered (averaged) to yield a system bandwidth of 2 Hz. Typical resolution tests are shown in Figure 6. The smallest difference in concentration of dissolved oxygen between the prepared test samples, which we were able to prepare and measure reliably and repeatably by the reference instrument, corresponded to 0.1 mg/L. As shown in Figure 6, an interchange of these test samples at the input of the proposed system caused an output change well above the proposed system’s noise level, thus the proposed system’s resolution exceeded 0.1 mg/L significantly. The estimated root-mean-square noise amplitude at the system output corresponded to about 0.015 mg/L in the case of the [Ru(phen)_3_]Cl_2_ dye reagent, which also indicates an achievable system’s measurement resolution.

For a better, more detailed representation of estimated performances for the proposed system, we included a comparison of our system to some representative commercially available sensors on the market and to some solutions described in other literature. The compared models and solutions from literature are presented in Table 1, and the data taken into consideration were accuracy and response time.

## 4. Conclusions

This paper presented a microfluidic, capillary-based fluorescent chemical sensor system. While the proposed system could be applied to a variety of different fluorescent sensing systems, the experimental system was applied and demonstrated as a dissolved oxygen sensor. The consumption of reagent was only 17.5 nL/min, or 0.75 mL/month. Similarly, the sample consumption and waste generation were of the order of only 0.058 mL/min, or 2.5 L/month. The proposed system thus demonstrates very low reagent consumption, limited liquid waste generation, and straightforward connectivity with any fluidic system. On the other hand, the proposed system might allow the application of fluorescent liquid reagents that are difficult for efficient immobilization into solid sensing films or similar structures, have low fluorescent yields, are prone to light- or analyte-induced damages, or do not yield a reversible chemical reaction. Pure silica glass (used for the manufacture of silica capillaries) is known widely for its chemical durability and compatibility with different environments. Inspection of the system before and after extensive testing was performed, and did not show the formation of any measurable changes or silica capillary degradation.

The system also has a relatively short response time, which is determined predominantly by the travel time of the tested fluid through the sample delivery capillary, and the time required for the chemical reaction to take place between the reagent and the sample. In the current system, with a 50 cm long sample delivery capillary, the total response time was about 60 s, but the reduction of capillary lengths is straightforward if a response time reduction is required. The proposed system allows for the use of relatively high-excitation light powers, as the flow-through concept of the system reduces the probability of the appearance of bleaching, heating, or other unwanted effects on the fluorescent dye/reagent caused significantly by the excitation light. Furthermore, the relatively large light–sample interaction volumes are native to the proposed system. In the current experimental case, over a 1 mm long and more than a 0.1 mm wide volume of liquid was illuminated selectively by a high-intensity excitation light, while the light-collecting fiber was placed in the closest possible proximity to this excited volume, which resulted in a relatively high amplitude of fluorescent optical signals collected by the light-collection fiber. The latter enables low-noise, high-bandwidth signal detection, which yields a high measurement resolution, and allows for the application of dyes/reagents with short fluorescent lifetimes. This opens possibilities for a broad reagent selection [5,6,7,8,9,10,12,35,36,41]. For example, in the current system configuration we demonstrated a resolution of 0.025 mg/L in O_2_ in water, while using dye with a fluorescent decay time of about 400 ns. The proposed system also eliminates the need for complex packaging that is typical for lab-on-a-chip and similar solutions. There are also a variety of possibilities for further improvement of the proposed system. For example, capturing of fluorescent light could be increased further by the application of higher NA light-collection fibers. Furthermore, a reduction of the APD detector diameter can be achieved by designing an in-fiber or on-detector filter, which would replace the bulk filter inserted in between the delivery fiber and the APD in the current experimental configuration. These improvements would result in an increased bandwidth, and/or improved signal-to-noise ratio, and, thus, system measurement resolution. A potential application of the proposed system for online measurements in industrial applications is, for example, in water or beverage preparation, and control in the food industry or wastewater monitoring. The system also supports the drinking water quality parameter monitoring listed in EU directives for waste and drinking water [46,47]. Different extensions of the proposed systems are also relatively straightforward. For example, by adding one more reagent delivery capillary, and, if required, light delivery fibers, to the existing setup (i.e., to the existing reagent mixing capillary), we could introduce additional reagents and excitation wavelengths into the measurement process simultaneously or time sequential, which would allow for simultaneous or quasi-simultaneous measurement of two or more analytes.

## Figures and Tables

**Table 1:** Comparison of commercially available [28] and other reported techniques of fluorescence-quenching oxygen sensor.

Company/Article	Product	Accuracy	Response Time
WTW	TriOxmatic		
	720 IQ FDO	±0.01 mg/L	30 s
	700 IQ FDO	±0.05 mg/L	<150 s
	701 IQ FDO	±0.1 mg/L	<60 s
Mettler	SG9	±0.01 mg/L	/
	SG6	±0.01 mg/L	90 s
HACH	LDOMT HQ10	±0.1 mg/L	<30 s
Sea-bird scientific	SBE 63	±0.1 mg/L	<6 s
YSI	EcoSense ODO200 ProSolo ODO	±0.15 mg/L ±0.1 mg/L (0–20 mg/L)	/ /
Kongsberg	CONTROS HydroFlash O_2_	±0.1 mg/L	<3 s
Chang-Yen, D.A.; Gale, B.K. [16]	/	0.6 mg/L	/
Onal, E.; Sass, S.; Hurpin, J.; Ertekin, K.; Topal, S.Z.; Kumke, M.U.; Hirel, C. [37]	/	6.3 Vol.%^−1^	(4.5 ± 0.5) s
McDonagh, C.; Kolle, C.; McEvoy, A.K.; Dowling, D.L.; Cafolla, A.A.; Cullen, S.J.; MacCraith, B.D. [34]	/	15 ppb	/
Zhang, H.L.; Zhang, Z.G. [21]	/	0.01 mg/L	(0.4 ± 0.2) s
This work	/	0.025 mg/L	<60 s

## Data Availability

Not applicable.

## Appendix A

**Table A1:** Chemicals employed in this investigation.

Chemical Name	Chemical Formula	CAS-No.	Supplier
Ruthenium-tris(2,2′-bipyridyl) dichloride	C_30_H_24_Cl_2_N_6_Ru·6H_2_O	50525-27-4	Sigma-aldrich
Dichlorotris(1,10-phenanthroline)ruthenium(II) chloride	C_36_H_24_Cl_2_N_6_Ru	23570-43-6	Sigma_aldrich
